# Identification of Merkel Cell Polyomavirus from a Patient with Acute Myeloid Leukemia

**DOI:** 10.1128/genomeA.01241-16

**Published:** 2017-01-19

**Authors:** Y. Song, P. Gyarmati

**Affiliations:** Department of Cancer Biology and Pharmacology, University of Illinois College of Medicine, Peoria, Illinois, USA

## Abstract

Merkel cell polyomavirus (MCPyV) is an oncogenic virus associated with Merkel cell carcinoma, an aggressive form of skin cancer with a high (>30%) mortality rate. The virus has a high incidence in patients with immunosuppressed conditions, such as AIDS or leukemia, or following organ transplantation. Here, we report the complete genomic sequence of MCPyV identified from a blood sample from a patient with acute myeloid leukemia.

## GENOME ANNOUNCEMENT

Merkel cell polyomavirus (MCPyV) is one of the 10 microorganisms designated carcinogenic by the International Agency for Cancer Research (1). The virus was first described in 2008, using a digital transcriptomic subtraction method (2). MCPyV is an icosahedral nonenveloped double-stranded DNA virus belonging to the *Polyomaviridae* family. The virus is associated with Merkel cell carcinoma, an aggressive form of skin cancer, and has a high incidence in immunosuppressed patients, especially in those with AIDS or chronic lymphocytic leukemia, and following organ transplants. The virus can also be found on healthy skin, causing no symptoms (3).

Here, we report the complete sequence of MCPyV identified in a blood sample from a 48-year-old male patient with acute myeloid leukemia. Sequencing was performed using a HiSeq 2500 instrument (4), and the viral sequence was assembled using RTG Core 3.4, resulting in 64× average coverage. Our assembly showed >99.9% identity to the reference strain NC_010277.2 (gi|733573629), with five mutations (A1203G, C1213T, G2034C, T2088A, and A3190G) detected. The mutations resulted in two amino acid changes (P405S and N1064D) but did not affect cleavage sites (5), and there were no changes in open reading frames.

### Accession number(s).

The whole-genome sequence has been deposited in GenBank using BankIt under accession number KX827417.

## References

[1] Garrett WS 2015 Cancer and the microbiota. Science 348:80–86. doi:10.1126/science.aaa4972. 25838377 PMC5535753

[2] Feng H, Taylor JL, Benos PV, Newton R, Waddell K, Lucas SB, Chang Y, Moore PS 2007 Human transcriptome subtraction by using short sequence tags to search for tumor viruses in conjunctival carcinoma. J Virol 81:11332–11340. doi:10.1128/JVI.00875-07. 17686852 PMC2045575

[3] Spurgeon ME, Lambert PF 2013 Merkel cell polyomavirus: a newly discovered human virus with oncogenic potential. Virology 435:118–130. doi:10.1016/j.virol.2012.09.029. 23217622 PMC3522868

[4] Gyarmati P, Kjellander C, Aust C, Song Y, Öhrmalm L, Giske CG 2016 Metagenomic analysis of bloodstream infections in patients with acute leukemia and therapy-induced neutropenia. Sci Rep 6:23532. doi:10.1038/srep23532. 26996149 PMC4800731

[5] Theiss JM, Günther T, Alawi M, Neumann F, Tessmer U, Fischer N, Grundhoff A 2015 A comprehensive analysis of replicating Merkel cell polyomavirus genomes delineates the viral transcription program and suggests a role for mcv-miR-M1 in episomal persistence. PLoS Pathog 11:e1004974. doi:10.1371/journal.ppat.1004974. 26218535 PMC4517807

